# Nanophytomedicines for the Prevention of Metabolic Syndrome: A Pharmacological and Biopharmaceutical Review

**DOI:** 10.3389/fbioe.2020.00425

**Published:** 2020-05-14

**Authors:** Zeinab Nouri, Marziyeh Hajialyani, Zhila Izadi, Roodabeh Bahramsoltani, Mohammad Hosein Farzaei, Mohammad Abdollahi

**Affiliations:** ^1^Students Research Committee, Faculty of Pharmacy, Kermanshah University of Medical Sciences, Kermanshah, Iran; ^2^Pharmaceutical Sciences Research Center, Health Institute, Kermanshah University of Medical Sciences, Kermanshah, Iran; ^3^Department of Traditional Pharmacy, School of Persian Medicine, Tehran University of Medical Sciences, Tehran, Iran; ^4^PhytoPharmacology Interest Group, Universal Scientific Education and Research Network, Tehran, Iran; ^5^Medical Biology Research Center, Kermanshah University of Medical Sciences, Kermanshah, Iran; ^6^Toxicology and Diseases Group, Pharmaceutical Sciences Research Center, The Institute of Pharmaceutical Sciences, Tehran University of Medical Sciences, Tehran, Iran; ^7^Department of Toxicology and Pharmacology, School of Pharmacy, Tehran University of Medical Sciences, Tehran, Iran

**Keywords:** medicinal plants, nanoparticles, diabetes, metabolic syndrome, nanophytomedicines, phytotherapy

## Abstract

Metabolic syndrome includes a series of metabolic abnormalities that leads to diabetes mellitus and cardiovascular diseases. Plant extracts, due to their unique advantages like anti-inflammatory, antioxidant, and insulin sensitizing properties, are interesting therapeutic options to manage MetS; however, the poor solubility and low bioavailability of lipophilic bioactive components in the herbal extracts are two critical challenges. Nano-scale delivery systems are suitable to improve delivery of herbal extracts. This review, for the first time, focuses on nanoformulations of herbal extracts in MetS and related complications. Included studies showed that several forms of nano drug delivery systems such as nanoemulsions, solid lipid nanoparticles, nanobiocomposites, and green-synthesized silver, gold, and zinc oxide nanoparticles have been developed using herbal extracts. It was shown that the method of preparation and related parameters such as temperature and type of polymer are important factors affecting physicochemical stability and therapeutic activity of the final product. Many of these formulations could successfully decrease the lipid profile, inflammation, oxidative damage, and insulin resistance in *in vitro* and *in vivo* models of MetS-related complications. Further studies are still needed to confirm the safety and efficacy of these novel herbal formulations for clinical application.

## Introduction

Metabolic syndrome (MetS), also known as “syndrome X” and “insulin-resistance syndrome,” is characterized by several metabolic abnormalities, including insulin resistance, type 2 diabetes, obesity, hypertension, and dyslipidemia (Kaur, 2014; Dalvand et al., 2017; Ebrahimi-Mameghani et al., 2018). About 20–30% of the world population is diagnosed with MetS, which makes the disease as a global health issue (Beltrán-Sánchez et al., 2013; Xi et al., 2013; Vishram et al., 2014; Pucci et al., 2017). MetS is the result of a series of genetic and environmental factors; however, the exact etiology is not yet understood (Feldeisen and Tucker, 2007). The underlying mechanisms encompass insulin resistance, elevated plasma free fatty acids, chronic inflammation, and oxidative stress (Bergman et al., 2001; Pan and Kong, 2018). The increased level of free fatty acids results in suppression of insulin clearance and is closely associated with insulin resistance in obese individuals. To overcome the resistance, pancreas secretes more insulin, leading to hyperinsulinemia (Oh et al., 2018). Free fatty acids cause induction and suppression of protein kinase in the liver and the muscle cells, respectively, which subsequently increases gluconeogenesis in liver and diminishes glucose uptake in muscles (Rochlani et al., 2017). Chronic inflammation is implicated in visceral obesity and exacerbates insulin resistance, which is characterized by the abnormal production of adipocytokines such as tumor necrosis factor-α (TNF-α), interleukin-1 (IL-1), IL-6, leptin, and plasminogen activator inhibitor-1 (PAI-1) (Vaziri et al., 2005; Di Lorenzo et al., 2013). Oxidative stress induces insulin resistance and also abrogates the adiponectin production by adipocytes (Furukawa et al., 2017). Adiponectin is an important anti-inflammatory and anti-atherogenic adipokine and is considered as a protective factor against the development and progression of chronic diseases related to metabolic disorders and oxidative stress including diabetes, hypertension, and cardiovascular diseases (Becic et al., 2018).

**FIGURE 1:**
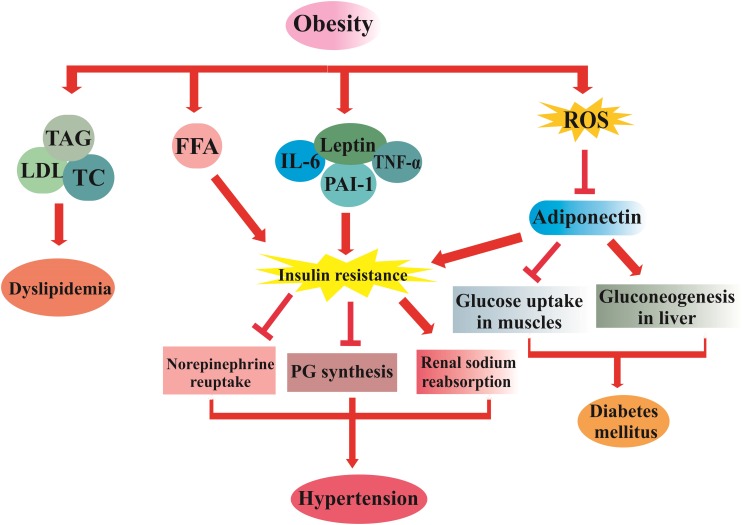
The pathophysiological mechanism of MetS. TNFα, tumor necrosis factor; PAI-1, plasminogen activator inhibitor-1; IL-6, interleukin 6; FFA, free fatty acid; PG, prostaglandin; TAG, triacylglycerol; LDL-C, low density lipoprotein; TC, total cholesterol; ROS, reactive oxygen species.

Secretions of adipose tissue stimulate mineralocorticoid release from adrenal cells and promote the renin angiotensin aldosterone system activity. Consequently, an elevation in renal sodium retention and vascular tone, as well as an inhibition of norepinephrine reuptake occur, which leads to hypertension. So, there is a direct relationship between obesity and the pathogenesis of hypertension (Ehrhart-Bornstein et al., 2003; Cabandugama et al., 2017). The pathophysiological mechanisms of MetS are schematically summarized in Figure 1. Management of MetS involves lifestyle modification, which consists of particular recommendations on physical activity and dietary interventions to achieve a normal weight, modulation of glycemic and lipid profile, as well as a decrease in blood pressure (Grundy, 2016).

**FIGURE 2:**
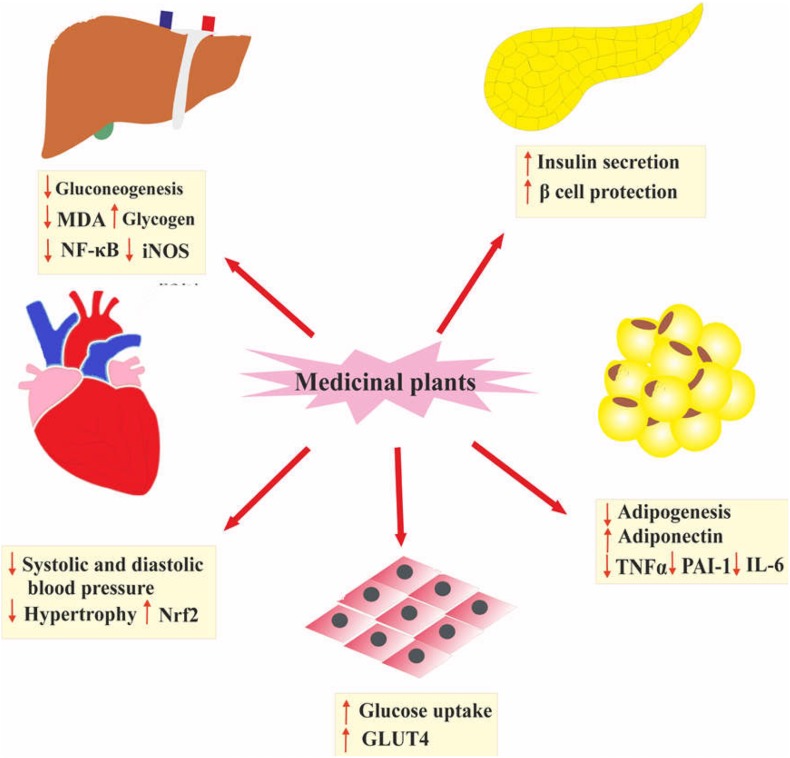
The main mechanisms of medicinal plants for managing MetS. Nrf2, nuclear factor erythroid 2–related factor 2; GLUT4, glucose transporter type 4; TNFα, tumor necrosis factor; PAI-1, plasminogen activator inhibitor-1; IL-6, Interleukin 6; MDA, malondialdehyde; NF-κB, nuclear factor-κB; iNOS, inducible nitric oxide synthase.

Several studies have shown that medicinal plants and their isolated compounds possess beneficial therapeutic effects like anti-inflammatory, antioxidant, and insulin-sensitizing properties (Naseri et al., 2018). Oral administration of plant extracts is shown to be effective on MetS and its complication by reducing visceral obesity, systolic and diastolic blood pressure, and blood glucose. Additionally, medicinal plants enhance insulin secretion and cardiovascular function, and suppress gluconeogenesis, inflammation and oxidative stress (Naseri et al., 2018; Payab et al., 2019). The main mechanisms of medicinal plants for managing MetS are presented in Figure 2. In spite of the promising effects of medicinal plants to manage MetS and its complications, due to a low bioavailability, their bioactivity is diminished (El-Far et al., 2016; Hassani et al., 2016; Cardozo et al., 2018; Firouzi et al., 2018; Hosseini et al., 2018; Tajmohammadi et al., 2018). One of the promising ways for the improvement of bioavailability, stability, solubility, and biodistribution of natural products is formulating these compounds in nanostructured forms (Gera et al., 2017).

Various nanostructured formulations such as green-synthesized nanoparticles (NPs), nanoemulsions, solid lipid nanoparticles (SLNs), nanostructured lipid carriers (NLCs), nanoliposomes, and polymeric NPs have been developed using natural products (Taghipour et al., 2019). Plant extracts mediated synthesis of NPs is one of the best and environment friendly method for the green synthesis of metal NPs (Khan et al., 2017).

Nanoemulsions are stable colloidal systems that are favorable and suitable vehicles for controlled delivery of lipophilic ingredients (Aswathanarayan and Vittal, 2019). SLNs are lipid-based NPs that can be easily fabricated by biodegradable and biocompatible solid lipids (Ghasemiyeh and Mohammadi-Samani, 2018). NLCs are another type of lipid-based nano carrier systems with colloidal particles composed of both solid and liquid lipids (Madane and Mahajan, 2016). Nanoliposomes provide a useful technology for delivering and targeting both hydrophilic and lipophilic phytobioactive constituents (Khorasani et al., 2018). Biodegradable polymeric NPs offer numerous advantages, since they protect bioactive constituents from degradation, enhance solubility, and provide controlled delivery and targeting (Pereira et al., 2018).

Ganesan et al. (2017) reviewed the beneficial effects of nanostructured formulations of phytochemicals to counteract diabetes. In our previous study, we reviewed the beneficial effects of nanoformulation originated from phytochemicals to combat MetS and its related complications (Taghipour et al., 2019). There is no comprehensive review about the potential use of various nanostructured formulations fabricated from herbal extracts, as promising future drugs to treat MetS and its associated complications. The present study, for the first time, provides a comprehensive review on the beneficial effects of nanoformulated herbal extracts on MetS and related disorders considering the *in vitro* and *in vivo* experiments.

## Nano Drug Delivery Systems for Metabolic Syndrome

Administration of nano-based drug delivery systems is one of the main strategies to enhance targeting capability and also to improve the safety and efficacy of drugs (Kesharwani et al., 2018). Conventional drug delivery systems are often accompanied with some critical limitations such as high dosage, low efficacy, low bioavailability, lack of target specificity, and dose-dependent side effects (Surendiran et al., 2009; Subramani et al., 2012). *In vitro* and *in vivo* investigations showed that nano-drug delivery systems such as nanomicelles, liposomes, and hydrogel-based nanocomposites can provide drug targeting to a specific site (Ponnappan and Chugh, 2015; Kesharwani et al., 2018). In case of diabetes and diabetes-associated dysfunctions, effective delivery of insulin via oral route by these nanoformulations is highly preferred compared to the available parenteral preparations, due to a higher patient compliance (Wilczewska et al., 2012; Fangueiro et al., 2015; Maity et al., 2017). In spite of the recent advancements of insulin delivery by NPs, there is still a challenge regarding the low bioavailability and poor gastric absorption of insulin. Some strategies have been utilized to overcome this challenge. For example, liposomes are mostly used to entrap lipophilic and hydrophilic drugs, to achieve higher efficacy and bioavailability, as well as fewer side effects. Liposomes modified with targeted ligand biotin were found to be effective in facilitating the delivery of insulin within oral route with limited leakage of insulin from inner aqueous parts and also facilitated insulin uptake through intestinal epithelia by receptor-mediated endocytosis (Zhang et al., 2014). The nanoliposomes had a longer gastric residence due to a higher resistance to enzymatic degradation by the proteolytic enzymes such as pepsin and trypsin. Conjugation of NPs with cell-penetrating peptides is another solution to improve the bioavailability (Wu et al., 2004). For example, peptide-protamine was used by Sheng et al. (2016), conjugated with insulin and encapsulated in poly (lactic-co-glycolic) acid (PLGA) NPs (as a mucoadhesive nanoformulation), and coated with *N*-trimethyl chitosan chloride. After oral administration of the formulation to diabetic rats, the onset of hypoglycemic effect was found to appear faster and remained longer. The bioavailability of this formulation was considerably increased, corroborating that the prepared nanoformulation could be internalized in cells easier than insulin (Sheng et al., 2016). NPs can also be coated with some protective agents such as chitosan, to prevent or decrease enzymatic digestion (Wu et al., 2004). Also, their mucus layer permeation can be promoted using coatings like *N*-(2-hydroxypropyl) methacrylamide copolymer (pHPM) (Qu et al., 2016).

SLNs are other nano-drug delivery systems which can potentially enhance the intestinal absorption and protect both peptide-based and organic hypoglycemic agents against enzymatic degradation (Sarmento et al., 2007).

Conclusively, nanoformulation of conventional drugs used for the management of MetS results in improved efficacy/bioavailability and reduced side effects; thus, the same approach can be employed to enhance delivery of plant-derived natural compounds to manage MetS which is discussed as follows.

## Nano Drug Delivery Systems Based on Herbal Extracts to Manage Different Types of Metabolic Disorders

### Diabetes Mellitus

Diabetes mellitus is a metabolic disorder which is caused by insulin resistance or progressive pancreatic beta cell failure and lack of insulin secretion, which cause a disturbance in the metabolism of carbohydrates, lipids, and proteins that consequently leads to micro- and macrovascular complications (Choudhari et al., 2017). Various plant extracts have been introduced to control diabetes and its complications. Nanostructured formulations of herbal extracts can potentiate their antidiabetic properties through the regulation of pharmacokinetics and increment of bioavailability (Ganesan et al., 2017). NLCs are drug delivery systems containing both solid and liquid lipids as a core matrix and can be used as drug carriers for lipophilic drugs, to increase their solubility and bioavailability (Ong et al., 2016). Marrubiin, a diterpenoid in the *Leonotis leonurus* (L.) R.Br., has a preventive and therapeutic effect in diabetes in experimental studies (Nakhlband et al., 2018). Marrubiin can induce insulin secretion, increase insulin sensitivity and upregulate *glucose transporter type (GLUT)-2* gene (Mnonopi et al., 2012). This phytochemical is poorly soluble in water. NLCs based on acetonic leaf extract of *L. leonurus* were fabricated by high-pressure homogenization method and were evaluated regarding antidiabetic effects in an *in vitro* model. Under hyperglycemic condition, the nanoformulation stimulated insulin release in INS1 pancreatic β cells and elevated glucose uptake in Chang liver cells compared to the extract. Extract-loaded NPs had an average particle size of 220 nm and were stable in different storage temperatures (Odei-Addo et al., 2017). Therefore, NLCs might be a suitable candidate to be evaluated in an animal model of diabetes.

*Ficus religiosa* L., commonly known as Peepal tree, possesses several pharmacological effects including antioxidant, anti-inflammatory, antidiabetic and neuroprotective activity (Singh et al., 2011). SLNs, formulated using an ethanolic stem bark extract of *F. religiosa*, were assessed in streptozotocin (STZ) and fructose-induced animal model of diabetes. Results showed that extract loaded SLNs had pronounced hypoglycemic and insulin sensitizing effects compared with the extract suspension. The nanoformulations based on SLNs provided an initial burst release followed by sustained release (Priyanka et al., 2018). SLNs offer attractive properties including easy production, low particle size, low toxicity, and good loading capacity of active molecules (Gordillo-Galeano and Mora-Huertas, 2018). In spite of various advantages of SLNs, the initial burst release makes the SLNs delivery systems unfavorable for oral delivery of several natural products that can improve chronic diseases (Ganesan et al., 2018). One of the promising ways to overcome this drawback is surface modification of the SLNs (Ganesan et al., 2018). Therefore, *F. religiosa* extract loaded surface modified SLNs can be the subject of future studies.

*Plicosepalus acaciae* (Zucc.) Wiens & Polhill and *P. curviflorus* (Benth. ex Oliv.) Tiegh. are medicinal plants which demonstrated antidiabetic activity, probably due to their antioxidant effects (Al-Taweel et al., 2012). Three SLNs based on methanolic extract of *P. acacia* and *P. curviflorus* were prepared with emulsion solvent evaporation method and their antidiabetic activity was evaluated in STZ+ high-fat diet (HFD)-induced diabetes. It was revealed that the proportion of lipid used in NPs directly correlates with pharmacological activity, so that the formulation with higher content of lipids had a better effect to reduce blood glucose, insulin resistance, and glycated hemoglobin compared with the simple extract or pioglitazone. Also, a remarkable decrease in malondialdehyde (MDA), as well as an increase in the endogenous antioxidant mediators, including glutathione (GSH), superoxide dismutase (SOD), and catalase (CAT) were observed with the SLNs having the highest lipid ratio (Aldawsari et al., 2014). A further area of research is needed to elucidate the hypoglycemic mechanisms of *P. acaciae* and *P. curviflorus* extracts-loaded SLNs formulations.

Alpha-eleostearic acid is a typical conjugated trienoic fatty acid isomer of conjugated linolenic acid (Tsuzuki et al., 2006). Previous research demonstrated the presence of a high level of α-eleostearic acid in bitter gourd (*Momordica charantia* L.) seed oil, which displays strong antioxidant and anti-inflammatory activity in an animal model of diabetes (Saha and Ghosh, 2012). The bioavailability and efficacy of α-eleostearic acid is low due to slow transport across the gastrointestinal (GI) tract (Plourde et al., 2006). Nanoemulsion colloidal systems can increase GI absorption of bioactive hydrophobic molecules (Kumar Dey et al., 2012). Nanoemulsion containing bitter gourd oil diminished blood sugar and enhanced antioxidant enzymes (CAT, SOD, and glutathione peroxidase (GPx) in alloxan (ALX)-induced diabetic rats. The nanoemulsion system in comparison to the conventional emulsion showed higher physical stability and remained mono-phasic during 12 weeks of storage, which is one of the advantages of nano-sized droplets of the emulsion. This nanoformulation improved bioavailability and decreased the required dose (Paul et al., 2014).

*Syzygium cumini* (L.) Skeels. [Synonym: *Syzygium jambolanum* (Lam.) DC.] is an Indian medicinal plant with previously demonstrated antidiabetic activity (Samadder et al., 2011). A high content of polyphenols in leaf extract of *S. cumini* is responsible for antidiabetic and anti-inflammatory effects (Ajiboye et al., 2018). The hypoglycemic effect of *S. cumini* is related to upregulation of GLUT4, phosphatidylinositol 3 kinase (PI3K), and peroxisome proliferator activator receptor gamma (PPAR-γ) (Anandharajan et al., 2006). The ameliorative effects of *S. cumini* can be augmented by encapsulation in polymeric NPs. PLGA, a biodegradable and biocompatible polymer made from lactic acid and glycolic acid monomers (Danhier et al., 2012), was used to prepare *S. cumini* nanoformulation by the solvent displacement method. The prepared nanoformulation, caused a significant increase in glucose uptake, glucokinase activity, and GLUT4 protein expression in L6 rat skeletal muscle cells. Additionally, reactive oxygen species (ROS) production, nuclear factor-κB (NF-κB), a key contributor to cellular inflammatory cascades (Baker et al., 2011), and inducible nitric oxide synthase (iNOS), the enzyme that synthesizes NO, were significantly reduced in the *in vitro* model. In the rat model of arsenic-induced diabetes, blood sugar and glycosylated hemoglobin levels in the extract- and nanoformulation-treated groups were significantly decreased, but the decrease by nanoformulation was more remarkable than that of the simple extract. The authors claimed that formulating this extract in form of NPs could improve its penetration into blood brain barrier (Samadder et al., 2012). Considering the central nervous system (CNS) complications of diabetes, such antidiabetic formulation may have a dual action to control both hyperglycemia and CNS effects of the disease which can be the subject of future studies.

Recently, a growing attention has been payed to the use of herbal extracts to produce metal-based biocompatible NPs due to its cost-effectiveness and eco-friendly nature, as well as high efficacy and stability (Ovais et al., 2016). Active phyto-constituents, adhered to metal NPs, are responsible for their reduction and stabilization (Mittal et al., 2013).

*Argyreia nervosa* (Burm. f.) Bojer, from the family Convolvulaceae has been used in traditional Indian medicine for several therapeutic indications such as antidiabetic, anti-inflammatory and diabetic wound healing (Singhal et al., 2011; Paulke et al., 2013). Silver NPs (AgNPs) prepared using an aqueous leaf extract of *A. nervosa* showed *in vitro* inhibitory effects on α-amylase and α-glucosidase, which are important enzymes in carbohydrate metabolism with IC_50_ values of 55.5 and 51.7 μg/ml, respectively. Adherence of the functional groups of the phytochemicals to AgNPs enhanced surface area and significantly increased the entrapment of free radicals compared with the simple extract (Saratale et al., 2017). Thus, the formulation may be a suitable candidate to be evaluated in an animal model of diabetes.

*Eysenhardtia polystachya* (Ortega) Sarg. is commonly known as “palo azul” and has shown beneficial effects on the alleviation of bladder disorders and kidney stone (Perez et al., 1998). According to previous studies, flavonoid enriched *E. polystachya* extract has diminished oxidative damage in an animal model of diabetes (Perez-Gutierrez et al., 2016). Campoy et al. biosynthesized Ag NPs using a hydromethanolic extract of *E. polystachya* bark. The nanoformulation elevated pancreatic β cells survival and ameliorated insulin resistance and hyperglycemia, as well as dyslipidemia in glucose-induced diabetes in zebrafish. Also, in INS1 pancreatic β cell line intoxicated with hydrogen peroxide (H_2_O_2_), nanoformulated extract could significantly restore the insulin secretory ability of cells, which indicates antidiabetic activity of extract to be, at least in part, attributed to its antioxidant properties (Campoy et al., 2018).

*Pouteria sapota* (Jacq.) with the common name of “sapote” is found in Mexico and South America. The highest concentration of polyphenols in the fruit of this plant are responsible for its antioxidant activity (Ma et al., 2004). AgNPs were green-synthesized using the aqueous leaf extract of *P. sapota* and evaluated regarding the antidiabetic activity in cellular and animal models. *In vitro* antidiabetic properties of the AgNPs was corroborated by decreasing non-enzymatic glycosylation of hemoglobin, inhibition of α-amylase, and enhancement of glucose uptake by yeast cells. In STZ-induced animal model of diabetes, biosynthesized AgNPs and extract significantly improved SOD and CAT activity, decreased blood glucose, and enhanced plasma insulin level (Prabhu et al., 2018). *P. sapota* extract and its biosynthesized AgNPs can be considered as an effective agent in the management of diabetes.

Some preclinical and clinical studies found that cinnamon (the genus *Cinnamomum* spp.) can improve insulin resistance, decrease blood glucose concentrations and hemoglobinA1c (HbA1c) (Cao et al., 2007; Crawford, 2009). *Cinnamomum litseifolium* Thwaites was collected from two different regions in India and the antidiabetic effects of the leaf essential oil, formulated as nanoemulsions, were evaluated. The essential oil sample, containing β-phellandrene as the major component (66%), showed a higher inhibitory effect on α-amylase and α- glucosidase enzymes rather than the other sample with methyl eugenol, (*E*)-caryophyllene, epi-α-muurolol, α-cadinol, and shyobunol as the main ingredients (totally constitute about 60% of the essential oil). It should be mentioned that both nanoformulations showed lower inhibition compared to acarbose (Sriramavaratharajan and Murugan, 2018). Further *in vivo* investigations are needed to confirm the efficiency of these preparations as hypoglycemic agents.

*Costus speciosus* (J. Koenig) Sm. (synonym *Cheilocostus speciosus* (J. Koenig) C. D. Specht) has shown an antidiabetic effect via induction of insulin secretion and improvement in insulin sensitivity (Ali et al., 2014). Ethanolic leaf extract of *C. speciosus* was loaded in PLGA NPs to enhance bioactivity and provide sustained release of active constituents. PLGA NPs increased the expression of *insulin (I&II)*, and *GLUT4* genes; while decreased the expression of *GLUT2* gene. In addition, the nanoformulation diminished blood sugar, enhanced high density lipoprotein cholesterol (HDL-C) and decreased total cholesterol (TC), triacylglycerol (TAG), and low density lipoprotein (LDL-C) in STZ-induced diabetic rats. The nanoformulated extract was more effective in controlling the lipid profile and blood glucose in comparison to the simple extract (Alamoudi et al., 2014).

Stevioside is a glycoside isolated from *Stevia rebaudiana* (Bertoni). Both *in vitro* and *in vivo* studies have indicated that stevioside and the extract of *S. rebaudiana* can be used as an alternative treatment for diseases associated with the MetS (Singla et al., 2017a; Ahmad and Ahmad, 2018). To provide a continuous-release formulation, titanium dioxide (TiO_2_) nanomaterial is a suitable choice due to the numerous pores which supplying a controlled release preparation (Lopez et al., 2010; López et al., 2011). Hydroethanolic leaf extract of *S. rebaudiana* was formulated using TiO_2_ nanomaterial by sol-gel method and was evaluated in ALX-induced diabetic rats. After 31 days of treatment, there was a significant and permanent decrease in blood glucose, glycosylated hemoglobin, TC, and TAG, along with a higher insulin concentration (*P* < 0.01) in animals treated with extract-loaded nanomaterial (20 and 30 μM) compared to the diabetic group treated with TiO_2_ nanomaterial alone. TiO_2_ nanomaterial could provide a sustained and controlled release of the plant extract which resulted in prolonged stimulation of insulin secretion (Langle et al., 2015). The previous study has shown nanoencapsulated stevioside in pluronic-F-68-poly-lactic acid (PLA) interestingly increased intestinal absorption, bioavailability, and provided a controlled release of the active component (Barwal et al., 2013). Perumal et al. (2016) have investigated hydromethanolic leaf extract of *S. rebaudiana*-loaded chitosan NPs prepared through the nanoprecipitation method. Chitosan is an eco-friendly, non-toxic and cost-effective polymer which is used as an excipient for drug delivery (Yadav et al., 2019). In the STZ-induced diabetic rats treated with the extract-loaded NPs, a considerable body weight gain and increase in SOD, CAT, and GSH, as well as a decrease in LPO were observed. *In vivo* antidiabetic evaluation showed a remarkable decrease in blood sugar and HbA1c in the group treated with nanoformulated extract (Perumal et al., 2016).

Gymnemic acid is the main active constituent of *Gymnema sylvestre* (Retz.) R. Br. ex Sm. with antidiabetic properties (Ota and Ulrih, 2017). Gold NPs (AuNPs), synthesized using the aqueous extract of *G. sylvestre*, significantly abated inflammatory mediators, including TNF-α, IL-6 and C-reactive protein in ALX induced diabetic animal model. In addition, lower blood glucose, HbA1c, TAG, LDL-C, as well as a higher HDL-C level were achieved in AuNPs-treated rats compared with the diabetic control group. The nanoformulated extract could also show some improvement in the histology of islet cells. It should be mentioned that there was no significant improvement in the insulin level and body weight in diabetic rats; therefore, the plant seems to have an insulin-stimulating effect rather than an insulin-mimetic effect in the slight improvement of insulin level (Karthick et al., 2014).

*Marsilea quadrifolia* L. is known as “aquatic fern” with antidiabetic and antioxidant activities (Zahan et al., 2011). Nanosized particles can penetrate efficiently into the cells and regulate its function. Biosynthesized AuNPs with methanolic extract of *M. quadrifolia* elevated glucose utilization in 3T3 L1 adipocytes, as an *in vitro* model of diabetes, in a dose depended manner. Moreover, the high dose of AuNPs (30 μg/ml) and pioglitazone showed a similar effect. Polyphenols and flavonoids of the extract attached to the surface of AuNPs improved bioavailability, induced transmitted GLUT4 vesicle to the cell membrane and probably increased glucose uptake in adipocytes (Chowdhury et al., 2017).

*Sambucus nigra* L. belongs to the Caprifoliaceae family. The fruit extract of *S. nigra* contains several polyphenols which are responsible for antidiabetic activity (Badescu et al., 2015). Biogenic AuNPs were formulated with acetonic fruit extract of *S. nigra*. There was no significant difference in insulin level between the STZ-induced diabetic rats treated with the extract or AuNPs compared with the diabetic control group. The extract failed to reduce blood sugar also. Although AuNPs reduced blood glucose, the change was not statistically significant. The biogenic AuNPs abolished expression of the cyclooxygenase (COX)-2, which produces prostaglandins that promote inflammation, and enhanced antioxidant activity in the diabetic animals. It can be concluded that the AuNPs can be considered as an adjuvant therapy in diabetes due to augmentation of antioxidant competence, as well as mitigation of inflammation (Opris et al., 2017).

*Vaccinium arctostaphylos* L. is a well-known medicinal plant in Iran. Previous studies indicated that several species of the genus *Vaccinium* have beneficial effects on diabetes (Cignarella et al., 1996; Martineau et al., 2006). Chemically synthesized ZnO NPs possess antioxidant, antimicrobial and antidiabetic activities (Das et al., 2013; Alkaladi et al., 2014; Nagajyothi et al., 2014). Functionalizing NPs with natural products can enhance their bioactivity and biocompatibility (Opris et al., 2017). Ethanolic fruit extract of *V*. *arctostaphylos* was used for green synthesis of ZnO NPs by microwave-assisted method. The biosynthesized ZnO NPs abolished fasting blood sugar and promoted HDL-C level. A remarkable decrease in TC was observed in the group treated with biogenic ZnO NPs. Overall, the biofabricated ZnO NPs revealed better treating efficacies on ALX-induced diabetes vs. the chemically synthesized ZnO NPs. Since both ZnO as well as the extract, have antidiabetic effects the synergistic effect caused greater efficiency of biogenic ZnO NP to control diabetes (Bayrami et al., 2018).

*Musa paradisiaca* L. is commonly known as “plantain” and belongs to the Musaceae family. Previous data showed a 38.13% decrease in blood glucose in animal treated with extract of unripe plantain (Eleazu et al., 2013). Stem juice extract of *M. paradisiaca* was used for green synthesis of AgNPs. A remarkable increase in insulin levels as well as a decrease in blood sugar and HbA1c was observed in the STZ-induced diabetic rats treated with AgNPs. Increase in glucose uptake and the induction of insulin secretion are the possible mechanisms responsible for these observations (Anbazhagan et al., 2017).

*Chamaecostus cuspidatus* (Nees & Mart.) C.D.Specht & D.W.Stev. is known as “insulin plant” with antidiabetic and antioxidant activities (Ponnanikajamideen et al., 2016). AuNPs green-synthesized using aqueous leaf extract of *C. cuspidatus* were evaluated in STZ-induced diabetic rats. AuNPs elevated body weight, decreased TC and abolished lipid peroxidation, hydroxyl radicals, and nitric oxide. Additionally, AuNPs slightly decreased blood glucose and increased glycogen and insulin level. Nanoformulation with the mentioned plant was cost-effective and reduced the required dose (Ponnanikajamideen et al., 2018). Active phyto-constituents adhered to metal NPs which are responsible for their reduction and stabilization need in-depth clarification in future studies.

Previous data have shown that the extract of *Hibiscus subdariffa* L. calyx (family Malvaceae), containing various polyphenols, can be potentially used for improving insulin resistance and diabetes-related nephropathy (Peng et al., 2014). ZnO NPs were formulated using an aqueous leaf extract of *H. subdariffa* prepared at 60 and 100°C and were investigated in STZ-induced diabetic mice. Remarkable decrease in blood sugar to 59.58 and 48.27% was observed in the group treated with ZnO NPs synthesized at 60 and 100°C, respectively. A lower level of pro-inflammatory cytokines (TNF-α, IL-1β, IL-6) which is associated with Th1, and a higher level of anti-inflammatory cytokines (IL-4 and IL-10) which is linked with Th2 (Charrad et al., 2016), was observed in the diabetic group treated with ZnO NPs produced at 60°C compared to 100°C. Moreover, ZnO NPs increased the expression of *glucokinase (GK)*, *insulin receptor A (IRA)*, and *GLUT2* genes as well as decreased the expression of *Pyruvate Kinase L/R* gene. Spherical-shape particles with lower size were observed in ZnO NPs synthesized at 60°C vs. 100°C. Generally, biosynthesized NPs at 60°C showed a higher antidiabetic effect vs. 100°C due to lower particle size, and the higher bioactive compounds used as a stabilizer (Bala et al., 2015).

ZnO NPs were synthesized using aqueous leaf extracts of different plants, such as *Azadirachta indica* A. Juss., *Hibiscus rosa-sinensis* L., *Murraya koenigii* (L.) Spreng, *Moringa oleifera* Lam, *Tamarindus indica* L., and were evaluated regarding antidiabetic and antioxidant activities in an *in vitro* model. All of the plants listed are medicinal plants which are widely used as traditional and folk remedies in India (Tachibana et al., 2001). The extract of these plants is rich in carbohydrates, glycosides, phenolic compounds, flavonoids, saponins, and tannins, with previously demonstrated antidiabetic and antioxidant activities (Kuppusamy et al., 2015). All of the extract-mediated ZnO NPs showed antioxidant effects, but the highest activity was observed with *T. indica* and *M. oleifera*, possibly due to the presence of proteins and amino acids that do not exist in other plants. ZnO NPs which were chemically prepared exhibited no antioxidant effect. Also, *T. indica* showed the highest α-amylase and α-glucosidase inhibition compared to ZnO NPs prepared with other plant extracts and chemically synthesized ZnO NPs (Rehana et al., 2017). Hence, phytosynthesized NPs seems to be a safe and efficient alternative for therapeutic use.

*Cassia Fistula* L. has shown antioxidant, antidiabetic and protective effect on diabetic nephropathy (Agnihotri and Singh, 2013). Biosynthesized AuNPs were prepared with aqueous stem bark extract of *C. fistula*. The extract is rich in lupeol, β-sitosterol, and hexacosanol that are responsible for the reduction of gold ions into AuNPs which occurs more quickly after adding the plant extract. Although oral administration of both extract and biosynthesized AuNPs significantly increased body weight and HDL-C level, and ameliorated blood glucose and HbA1c, the nanoformulation was more effective. Improvement of lipid profile probably occurred due to the presence of β-sitosterol in the extract. In addition, biogenic AuNPs were more effective in reduction of urea, creatinine, and uric acid level vs. extract alone. This result showed beneficial effects of biogenic AuNPs as a promising agent in the management of complications associated with diabetes (Daisy and Saipriya, 2012).

*Silybum marianum* L., belonging to Asteraceae family, is one of the oldest medicinal plants and has been widely used for treating liver and gallbladder illnesses (Kosina et al., 2019). Silymarin, extracted from the *S. marianum* fruits, is a multicomponent extract and has demonstrated antioxidant and antidiabetic activities (Heidari Khoei et al., 2019). ZnO NPs biosynthesized using *S. marianum* seed extract reduced fasting blood sugar, TC, TAG and augmented HDL-C and insulin level in ALX-induced diabetic rats. On the other hand, ZnO NPs exerted antibacterial activity against *Escherichia coli* (Arvanag et al., 2019). Similarly, ZnO NPs prepared by *Nasturtium officinale* R.Br. leaf extract exhibited antidiabetic and antibacterial performances against the most common bacteria found in diabetic foot ulcer including *Staphylococcus aureus* and *E. coli* (Aris et al., 2019; Bayrami et al., 2019). Therefore, future studies should evaluate the beneficial effect of ZnO NPs prepared by *S. marianum* and *N. officinale* against diabetic wound infection.

The ginger extracted from the *Zingiber officinale* is considered as an antidiabetic agent through interfering with NF-κB cascade pathway and displaying anti-inflammatory and antioxidant properties (Saedisomeolia et al., 2019). The application of nanotechnology provides a useful delivery system for ginger compounds and enhance their biological activities (Zhang et al., 2016, 2018). Nano transdermal delivery for ginger ingredient, gingerol, increased its bioavailability ratio (Baskar et al., 2012). In an *in vivo* study performed by Garg et al. (2016) fabricated AgNPs using ethanolic extract of ginger rhizome exhibited notable hypoglycemic properties. Further investigations are needed in order to understand the clarification of the molecular mechanisms of ginger extract based NPs on diabetes.

Altogether, several findings revealed that nanostructured formulations of plant extracts were able to overcome drawbacks of chemical counterparts, affecting the promising effects of plant extracts in diabetes. Considering the critical role of herbal-based nanoformulations in combating inflammation and oxidative damages, future studies should focus on other aspects of their uses in the management of oxidative and inflammation related diseases. Almost all of the current evidence focused on the efficacy of plant extracts-loaded NPs in *in vitro* and/or *in vivo* models of diabetes. Future preclinical and clinical trials should be conducted to confirm their safety and efficacy in diabetic patients. Toxicological aspects, plausible side effects as well as main molecular mechanisms of plant extracts-loaded NPs need appropriate assessment before conducting clinical trials. Additionally, more profound investigations and engineered methods are required to design surface modified nanostructures of plant extracts to achieve optimized drug delivery systems.

## Diabetic Wound Healing

Delayed diabetic wound healing is associated with impaired angiogenesis and bacterial infection. Also, delayed treatment of diabetic foot ulcer can lead to foot amputation (Muniandy et al., 2018). Hence, due to the advantages of medicinal plants for wound healing, natural product-based nanoformulations can be used for amelioration of the wound. A nanobiocomposite hydrogel was prepared as cellulose nanocrystals using *Dendrocalamus hamiltonii* Nees & Arn. ex Munro, and *Bambusa bambos* (L.) Voss with previously demonstrated antimicrobial and wound healing effects (Singla et al., 2017b). Cellulose nanocrystals were then impregnated with AgNPs and were evaluated in the excision wound in STZ-induced diabetic mice. The nanoformulation abolished the level of TNF-α and IL-6, which are suppressors of fibroblast proliferation and epithelialization (Dinh et al., 2012). In the last stage of wound healing, the nanobiocomposite enhanced the level of transforming growth factor β (TGF-β), which stimulates the formation of granulation tissue and increases wound closure rate (Wang et al., 2013). Additionally, nanoformulation increased the level of platelet-derived growth factor (PDGF), fibroblast growth factors (FGF), and vascular endothelial growth factor (VEGF), participating in wound healing process by induction of angiogenesis and epithelization (Losi et al., 2013). Nanoformulation-treated group elevated density of collagen fibril and promoted complete wound healing (98–100%). Both cellulose nanocrystals and AgNPs possess anti-inflammatory and antimicrobial effects. A synergistic effect caused faster wound healing. Also, cellulose nanocrystals create a moist environment and reduce wound healing time (Singla et al., 2017c). Nanobiocomposite hydrogel can be considered as a suitable dressing material for wound healing purpose and seems as a favorable choice for skin drug delivery system.

## Vascular Complications of Diabetes

Long-term hyperglycemia associated with inflammation and oxidative damage contributes to the development of diabetic complications such as retinopathy, neuropathy, nephropathy, and cardiovascular disease (Volpe et al., 2018).

The earliest manifestation of cardiomyopathy is cardiac diastolic impairment followed by systolic dysfunction that consequently leads to heart failure (Dai et al., 2018). Among various pathophysiological mechanisms, ROS-related hyperglycemia caused oxidative stress in cardiomyocytes, and seems to play a critical role in diabetic cardiomyopathy (Xu Z. et al., 2016). Herbal remedies can have an important role in the amelioration of diabetic cardiomyopathy. Atale et al. (2017) evaluated the cardioprotective effects of green synthesized AgNPs prepared by methanolic seed extract of *S. cumini* on H9C2 cells derived from embryonic rat heart. Both extract and biogenic AgNPs ameliorated the size of glucose-stressed cells and decreased lipid peroxidation, but the decrease by AgNPs was more remarkable than that of the extract. The methanolic extract of *S. cumini* seems to be rich of polyphenolic compounds with antioxidant activity (Neha et al., 2013). Both AgNPs as well as the extract have antioxidant properties; therefore, the synergistic effect caused greater efficiency of biogenic Ag NPs vs. extract (Atale et al., 2017).

Diabetic nephropathy (DN) is considered as a major cause of end stage renal disease. DN is characterized by progressive proteinuria and decrease in glomerular filtration rate resulting in the loss of renal function (Bhattacharjee et al., 2016). It has been demonstrated that pomegranate (*Punica granatum* L.) has promising therapeutic effects against diabetes, cardiovascular complications and DN (Zarfeshany et al., 2014; Shehab et al., 2018). Most of the phytobioactive compounds are unstable and susceptible to enzymatic/non-enzymatic hydrolysis (Volf et al., 2014). In spite of widespread promising effects, pomegranate peel extract has remained underused. AuNPs produced by pomegranate peel extract ameliorated STZ-induced glomerular sclerosis and renal fibrosis. From a mechanistic point of view, AuNPs abrogated pro-inflammatory cascade in nephritic tissue through the regulation of the MAPK/NF-κB/STAT3 pathway. Additionally, AuNPs increased antioxidant performance by activation of nuclear factor erythroid 2–related factor 2 (Nrf2). AuNPs diminished protein glycation through the suppression of RAGE- NOX-4/p47^phox^ activation followed by the mitigation of the ROS generation (Manna et al., 2019). Considering the results, the biogenic AuNPs prepared by pomegranate extract may be a desirable agent to combat DN.

Mulberry leaf (*Morus alba* L.) is a traditional medicine used for treating diabetes. Mulberry leaf extract displayed hypoglycemic and insulin sensitizing effects through the activation of IRS-1/PI3K/Glut-4 signaling pathway (Cai et al., 2016). Diabetic retinopathy is considered as a common complication of diabetes which caused visual dimming and blindness (Rübsam et al., 2018). Inflammation, oxidative stress and hyperglycemia play a key role in diabetes-mediating retinopathy (Yeh et al., 2016). AgNPs biosynthesized using mulberry leaf extract alleviated deterioration in the retinal cell layer in diabetes or Aluminum-intoxicated rats (Xu et al., 2019). Further mechanistic studies are needed to confirm the beneficial effect of AgNPs fabricated by mulberry leaf extract to combat diabetic retinopathy.

## Obesity

Obesity is a major global health concern and is characterized by an imbalance between food intake and energy expenditure which causes excessive accumulation of fat in blood, adipose tissue, and liver (de Freitas Junior and de Almeida, 2017; Lee et al., 2018). Obesity is an important risk factor for type 2 diabetes, cancer, and cardiovascular diseases (Grigoraş et al., 2018). Phytosome is a phyto-phospholipid complex, which is produced by forming a hydrogen bond between polar part of phospholipids and phytochemicals. Phytosome is more stable than liposome which results from the hydrogen bond formation (Ghanbarzadeh et al., 2016). Phytosome is considered as an important approach to overcome the poor absorption and low bioavailability of phyto-constituents (Chi et al., 2020). Soybean, *Glycine max* (L.), is an example of a plant that has been investigated as an alternative choice for common chemical anti-obesity medications due to its content of different bioactive peptides (Singh et al., 2014). Methanolic extract of soybean was used for the synthesis of nanosized phytosome-based thermogel and evaluated for pharmacological effects in HFD-induced obese rats. Solvent evaporation, co-solvency and salting out methods were used for the preparation of phytosomes, as well as a cold method for preparation of a thermogel. Thermogel possesses thixotropic behavior, i.e., the transient viscosity of the fluid depends on its shearing (Wei et al., 2019). The gel transformation temperature was optimized at 31.5°C. The nanophytosome reduced weight gain, adipose tissue weight and daily intake of food. In addition, topical treatment with nanophytosomal thermogel significantly reduced TC, TAG, LDL-C and very low-density lipoprotein (VLDL)-C compared to the negative control group. Nanosized particles and high skin penetration of soy extract-loaded phytosomes seem to be responsible for the systemic effect. Nanophytosome formulation of soybean caused better release (92.50% within 2 h), which has a direct and reverse relation with increased percentage of extract and phosphatidylcholine in the phytosome, respectively. The nanophytosome revealed high stability and perfect entrapment efficiency due to the formation of a hydrogen bond between OH groups in the extract and phosphatidylcholine in the phytosome (El-Menshawe et al., 2018). Therefore, it can be concluded that nanophytosomal thermogel of soybean may be a useful formulation in treatment of obesity. In another study, AuNPs prepared by aqueous extract of *Smilax glabra* Roxb. Decreased body weight, blood glucose, and liver marker enzymes in HFD and STZ-induced obese diabetic rats. From the mechanistic point of view, AuNPs alleviated inflammatory markers including TNFα and IL-β, abolished leptin and resitin, and increased adiponectin in obese diabetic rats (Ansari et al., 2019). From the results, AuNPs represented an outstanding performance in alleviating both diabetes and obesity. It could possibly be used in future as a promising agent to improve obesity-related complications.

## Dyslipidemia

Dyslipidemia is a serious metabolic disorder accompanied by lipid abnormalities such as elevated TAG, VLDL-C, TC, LDL-C and decreased HDL-C (Sun et al., 2018). Management of dyslipidemia is important since it is a risk factor for cardiovascular diseases (Buşilă et al., 2017). One possible mechanism by which dyslipidemia leads to cardiovascular disorders is the generation of free radicals and oxidative stress (Singh et al., 2016). Previous studies demonstrated the antioxidant and antihyperlipidemic potential of both garlic oil and kenaf seed oil (Ragavan et al., 2017; Cheong et al., 2018). Garlic (*Allium sativum* L.) oil mostly constitutes sulfur-containing compounds such as alliin, allicin, diallyl sulfide, diallyl disulfide, and diallyl trisulfide which are responsible for antidiabetic, antihyperlipidemic and anti-atherogenic effects (Zheng et al., 2013; Sambu et al., 2015; Ebrahimzadeh-Bideskan et al., 2016). The use of garlic oil is limited due to its strong odor, stomach irritation, low stability, poor solubility and bioavailability (Gao et al., 2013). Nanoemulsion of garlic oil was prepared with a proportion of 1:2 (oil: surfactant) and evaluated regarding its preventive and therapeutic effects on HFD-induced dyslipidemia in an animal model. In the preventive method, HFD and drugs (garlic oil, nanoformulation, and standard drug) were administrated simultaneously and at the end of the 6 weeks, serum biochemical parameters were measured; whereas in the curative method, HFD was administrated during 10 weeks and drugs (garlic oil, nanoformulation, and standard drug) were given during the last 6 weeks. Nano-sized droplets had a spherical shape and were stable without forming sedimentation or phase separation. In the animal study, body weight gain was significantly lower in nanoformulation, standard drug, and garlic oil-treated rats in comparison to HFD control rats. Garlic oil nanoemulsion reduced lipid profile dose-dependently in both preventive and curative studies. Dyslipidemia causes liver injury and induces alteration in the hepatic enzymes such as ALT, AST, and ALP (Cheraghpour et al., 2019). These markers were significantly increased in rats treated with atorvastatin, garlic oil and nanoformulation of garlic oil at a concentration of 0.65 mg/kg; while they were decreased in rats treated with nano-encapsulated garlic oil at concentrations of 0.18 and 0.46 ml/kg. Lymphocytic infiltration and necrosis, which indicates liver damage (Huang et al., 2019), were not observed in animals treated with 0.18 and 0.46 ml/kg of nanoemulsion. Hematoxylin and Eosin staining revealed a decrease in vesicular steatosis (presence of numerous vesicle of fat in the liver) at 0.65 ml/kg of the nanoformulation. Moreover, 0.18 and 0.46 ml/kg of nanoemulsion were more effective in prevention and treatment of hepatic steatosis induced by HFD. Nanoformulation of garlic oil had higher efficiency, stability, and lower liver toxicity than direct intake of garlic oil (Ragavan et al., 2017). This nanostructured formulation of garlic oil could be an advantageous formulation for treatment of dyslipidemia.

According to the previous studies, natural ingredients of Kenaf (*Hibiscus cannabinus* L.) seed oil have a cholesterol-lowering effect. On the other hand, because of its poor solubility in water, its bioavailability is low (Cheong et al., 2018). Kenaf seed oil was prepared as a nanoemulsion and a macroemulsion and was investigated in high cholesterol diet (HCD)-induced dyslipidemia in rats. Nanoemulsion showed higher stability than macroemulsion due to its smaller droplet size. Beta-cyclodextrin in the emulsifier mixtures has a cholesterol-lowering effect by inhibiting the absorption of cholesterol in the small intestine (García-Mediavilla et al., 2003). Also, natural compounds present in the kenaf seed oil such as phytosterols and saponins have cholesterol-lowering effect (Shi et al., 2004; Lu et al., 2010). Synergistic effect between phytochemicals of kenaf seed oil and sodium caseinate as well as beta-cyclodextrin caused the lowest level of TC and LDL-C in nanoemulsion treated rats. Moreover, prepared nanoemulsion alleviated atherogenic and coronary risk index and increased antioxidant performance corroborated by reducing MDA and elevating GSH. In addition, nanoemulsion diminished accumulation of fat droplets in the liver and enhanced binucleate cells, which shows that hepatic cells can be regenerated after damage. Body weight gain was remarkably decreased in emulsifier mixtures and nanoemulsion treated rats, while simple kenaf seed oil revealed the lowest effect on weight gain. Therefore, weight loss probably occurs due to the presence of sodium caseinate, as well as beta-cyclodextrin in the emulsifier mixtures. Casein is the main protein of milk that has been reported to have a weight loss effect (Anderson et al., 2007). Overall, this nanoformulating method increased the bioavailability, stability, and efficacy of kenaf seed oil (Cheong et al., 2018).

## Pulmonary Arterial Hypertension

Pulmonary arterial hypertension (PAH) is a progressive medical condition characterized by disturbance in the pulmonary vascular function, increase in vascular resistance and obstruction of the pulmonary artery, which eventually result in right ventricular hypertrophy as well as right-sided heart failure (Kikuchi et al., 2018). Oxidative stress, nitric oxide, and inflammation participate in the development of PAH (Xu et al., 2017). Antioxidant and anti-inflammatory effects of phytochemicals make them a promising option for treating PAH (Xu D. et al., 2016; Meghwani et al., 2018; Xiang et al., 2018). Copaiba oil is an oil-resin that is produced from different species of the genus of *Copaifera* and is used in Brazilian traditional medicine (Kanis et al., 2012). β-caryophyllene is the major constituent of copaiba oil with antioxidant and anti-inflammatory effects (Ames-Sibin et al., 2018). In addition, this compound is a calcium channel blocker and has an inhibitory effect on cell growth (Rasheed et al., 2015). Nanoencapsulated copaiba oil was investigated in Monocrotaline (MCT)-induced PAH. Both free oil and NPs enhanced sulfhydryl groups (SH), SOD, GPx, and Nrf2, as well as abolished oxidized glutathione (GSSG) concentration, but nanocapsule was more effective. Nrf2 is an antioxidant transcription factor that plays a pivotal role in the protection of cells against oxidative stress (Hu et al., 2019). Both oil and nanoformulation possessed significant effect on the decrease of right ventricular hypertrophy index. Treatment with free oil significantly increased acceleration time/ejection time ratio, which indicates a decrease of PAH; whereas nanocapsules had no significant effect on pulmonary vascular resistance. Thus, the authors concluded that nanoformulation is more effective on heart tissue than pulmonary circulation. Moreover, the preparation of nanocapsules using pectin aqueous solution with antioxidant effect could have a synergistic effect with copaiba oil and increased pharmacological effect (Campos et al., 2017). Therefore, nanoencapsulation has been known effective in favorable delivery and better efficiency of copaiba oil.

## Conclusion

**FIGURE 3:**
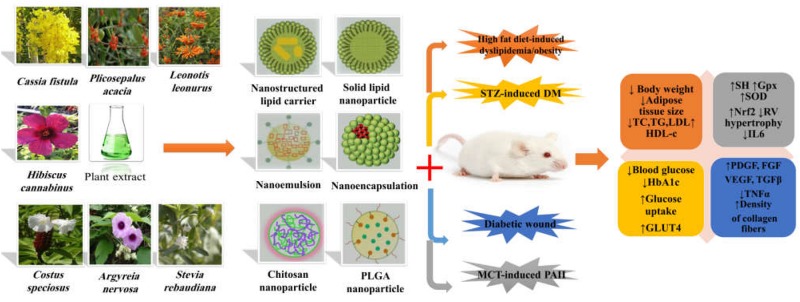
The role of extract-loaded nano-delivery systems in management of metabolic syndrome and its complications. STZ, streptozotocin; MCT, monocrotaline; PAH, pulmonary arterial hypertension; TC, total cholesterol; TAG, triacylglycerol; LDL-C, low-density lipoprotein-cholestrol; VLDL, very-low-density lipoprotein; HDL-C, high-density lipoprotein; SH, sulfhydryl group; GPx, glutathione peroxidase; SOD, superoxide dismutase; Nrf2, nuclear factor erythroid 2–related factor 2; RV, right ventricular; IL-6, interleukin 6; PDGF, platelet-derived growth factor; FGF, fibroblast growth factors; VEGF, vascular endothelial growth factor; TGFβ, transforming growth factor β; TNFα, tumor necrosis factor; GLUT4, glucose transporter type 4; HbA1c, hemoglobin A1c; PLGA poly lactic-co-glycolic acid.

Considering the complications of MetS and different mechanisms involved in its pathophysiology, successful strategies are necessary for prevention and treatment of the disease. Despite the evidence provided over the past decades regarding the therapeutic effects of the plant-derived compounds or herbal extracts on the quality of life and human health, their delivery is always problematic. In the last years, nano-based drug delivery systems have been introduced as one of the main strategies to overcome these problems to improve the efficiency of herbal extracts in the treatment of MetS and its related complications (Figure 3). Diabetes is recognized as a challenging metabolic disease and its management is always problematic due to its complexity. As presented, nanoformulations of herbal extracts as NLCs, SLNs, nanoemulsions colloidal systems, and other formulations have shown a significant increase in the antidiabetic effects of the extracts compared with the conventional formulations. Also, the type of nanoformulation and their preparation method had clearly a direct role in their antioxidant activity which depends on the chemical characteristics and the degree of solubility of these compounds. For example, nanoemulsions due to high stability can be a good candidate to deliver the hydrophobic extracts to improve bioavailability and decrease the required dose. On the other hand, the green synthesis of metal NPs such as Zn, Ag, and Au supports this conclusion that Au NPs can be more effective in improving diabetes-related complications.

In case of delayed diabetic wound healing, which is one of the complications of diabetic patients, the antibacterial properties of nano-formulated plant extracts have provided excellent advantages for improving diabetic wound healing and its associated problems. Considering the included studies in this review, cellulose based-biocomposites along with AgNPs showed the successful recovery even in the last stage of wound healing in a mouse model. Other characteristics of herbal extracts are antioxidant and anti-inflammatory properties, which make them promising therapeutic agents in the management of diabetic cardiomyopathy and obesity. Studies on the delivery of extracts with AgNPs were presented as great examples to decrease oxidative stress in cardiomyocytes and nanophytosome formulations in anti-obesity therapy.

Another unique property of medicinal plants, especially sulfur-containing herbal oils, is antihyperlipidemic potential which has demonstrated the highest effect on dyslipidemia in the nanoemulsion formulation. In addition, nanoemulsion and nanoencapsulation of these compounds demonstrated beneficial therapeutic effects on PAH.

Our purpose in the current review was to criticize a collection of pharmaceutical and biopharmaceutical studies on the effect of nanoformulation of plant extracts and comparison of different nanostructures such as lipid-based carriers (SLNs and NLCs), nanoemulsions and green synthesized metal NPs on metabolic disorders through *in vitro* and *in vivo* experiments (Table 1).

**TABLE 1:** Herbal extracts based nanoscale drug delivery systems for treating metabolic syndrome.

Plant	Nanoformulation Type	Disorder	Cellular/Animal model	Size	Outcome	References
*Leonotis leonurus*	Nanostructured lipid carrier	Diabetes mellitus	*In vitro* on INS1 pancreas β cell line and Chang liver cell.	220 nm	↑insulin sensitivity ↑Glucose uptake	Odei-Addo et al., 2017
*Plicosepalus acacia & P. curviflorus*	Solid lipid nanoparticles	STZ + HFD-induced diabetes mellitus	*In vivo* on Wistar rats	22–70 nm	↓insulin resistance ↑CAT↑SOD ↑GSH	Aldawsari et al., 2014
*Pouteria sapota*	Green synthesized Ag nanoparticle	STZ-induced Diabetes mellitus	*In vivo* on albino Wistar rats *In vitro* on yeast cells	–	↓blood glucose ↑serum insulin ↓ alpha amylase ↑glucose uptake	Prabhu et al., 2018
*Stevia rebaudiana*	chitosan nanoparticles	STZ-induced diabetes mellitus	*In vivo* on Wister rats	50.42–73.34 nm	↓FBS ↓HbA1c ↑SOD ↑ CAT ↑GSH	Perumal et al., 2016
*Syzygium cumini*	PLGA nano-encapsulated	Arsenic- induced diabetes mellitus	*In vitro* on L6 rat skeletal muscle cells *In vivo* on Swiss albino mice	122 nm	↑ glucose uptake ↑glucokinase ↑GLUT4 ↓ blood glucose, ↓NF-κB ↓ iNOS	Samadder et al., 2012
*Syzygium cumini*	Green synthesized Ag nanoparticle	Glucose- stressed cells	*In vitro* on Embryonic rat heart-derived H9C2 cells	40–100 nm	↓cell size ↓lipid peroxidation	Atale et al., 2017
*Eysenhardtia polystachya*	Green synthesized Ag nanoparticle	Glucose- induced diabetes mellitus	*In vivo* on Zebrafish *in vitro* on INS1 pancreas β cell line	5–21 nm	↓blood glucose ↓insulin secretion ↓TC	Campoy et al., 2018
*Musa paradisiaca*	Green synthesized Ag nanoparticle	STZ-induced diabetes mellitus	*In vivo* on albino Sprague Dawley rats	30–60 nm	↓blood glucose ↓HbA1c ↑insulin ↑glycogen	Anbazhagan et al., 2017
*Cassia fistula*	Green synthesized Au nanoparticle	STZ-induced diabetes mellitus	*In vivo* on male albino Wistar rats	55.2–98.4 nm	↓Blood glucose ↓HbA1c ↓ LDL-C ↑ HDL-C	Daisy and Saipriya, 2012
*Gymnema sylvestre*	Green synthesized Au nanoparticle	ALX–induced diabetes mellitus	*In vivo* on Wistar albino rats	Ave:50 nm	↓ TNFα↓IL6 ↓CRP ↓HbA1c ↓ LDL-C ↑HDL-C	Karthick et al., 2014
*Sambucus nigra*	Green synthesized Au nanoparticle	STZ-induced diabetes mellitus	*In vivo* on Wistar rats	4–23 nm	↓ blood glucose ↓MDA ↓ COX2	Opris et al., 2017
*Marsilea quadrifolia*	Green synthesized Au nanoparticle	Diabetes mellitus	*In vitro* on 3T3-L1 adipocytes	17–40 nm	↑ glucose utilization	Badescu et al., 2015
*Chamaecostus cuspidatus*	Green synthesized Au nanoparticle	STZ –induced diabetes mellitus	*In vivo* on albino rats	Ave: 50 nm	↓Blood glucose ↑body weight ↓super oxide anion ↓lipid peroxidation	Ponnanikajamideen et al., 2018
*Stevia rebaudiana*	TiO2 nanomaterial	ALX-induced diabetes mellitus	*In vivo* on Long Evans rats	Ave: 4 nm	↓blood glucose ↑insulin ↓HbA1c ↓TC ↓TAG	Langle et al., 2015
*Moringa oleifera*	Green synthesized ZnO nanoparticle	Diabetes mellitus	*In vitro*	Ave: 27.61	↓α-amylase ↓α-glucosidase.	Rehana et al., 2017
*Tamarindus indica*	Green synthesized ZnO nanoparticle	Diabetes mellitus	*In vitro*	Ave: 25.66	↓α-amylase ↓α-glucosidase.	Rehana et al., 2017
*Hibiscus subdariffa*	Green synthesized ZnO nanoparticle	STZ-induced diabetes mellitus	*In vivo* on Swiss albino mice	12-46 nm	↓TNF-a ↓IL-1b ↓IL-6 ↑ IL-4 ↑ IL-10 ↑GK ↑IRA ↑GLUT2 ↓ PKLR	Bala et al., 2015
*Vaccinium arctostaphylos*	Green synthesized ZnO nanoparticle	ALX-induced diabetes mellitus	*In vivo* on male Wistar rats	Ave: 13.9 nm	↓ blood sugar ↑HDL-C ↓TC	Bayrami et al., 2018
*Glycine max*	Nanophytosome	HFD-induced obesity	*In vivo* on male albino rats	51.66–667.24 nm	↓ body weight, ↓TC, LDL-C	El-Menshawe et al., 2018
*Allium sativum*	Nanoemulsion	HFD-induced dyslipidemia	*In vivo* on albino Wistar rats	20–46 nm	↓TC ↑ HDL-C ↓AST ↓ALT	Ragavan et al., 2017
*Hibiscus cannabinus*	Nanoemulsion	HCD-induced dyslipidemia	*In vivo* on Sprague–Dawley rats	133.4 nm	↓TC ↓ LDL-C ↑GSH ↓fat droplet in liver	Cheong et al., 2018
*Copaifera* sp.	Nanoencapsulated	MCT-induced Pulmonary arterial hypertension	*In vivo* on Wistar rats	139.03 nm	↑SH ↑Gpx ↑SOD ↑Nfr2 ↓RV hypertrophy	Campos et al., 2017
*Dendrocalamus hamiltonii*	Nanobiocomposite (cellulose nanocrystals and AgNPs)	Diabetic wound	*In vivo* on Swiss albino mice	Nano crystal: 18 ± 0.5 nm and AgNPs 16 ± 3 nm	↓TNFα↓IL6 ↑PDGF ↑FGF ↑VEGF ↑ TGFβ↑Density of collagen fibers	Singla et al., 2017c
*Bambusa bambos*	Nanobiocomposite (cellulose nanocrystals and AgNPs)	Diabetic wound	*In vivo* on Swiss albino mice	Nano crystal: 20 ± 1 nm and AgNPs: 22 ± 7 nm	↓TNFα↓IL6 ↑PDGF ↑FGF ↑VEGF ↑ TGFβ↑Density of collagen fibers	Singla et al., 2017c
*Argyreia nervosa*	Green synthesized Ag nanoparticle	Diabetes mellitus	*In vitro*	5–35 nm ave:15 nm	↓α-amylase ↓α-glucosidase	Saratale et al., 2017
*Cinnamomum litseifolium*	Nanoemulsion	Diabetes mellitus	*In vitro*	102.2 nm	↓α-amylase ↓α- glucosidase	Sriramavaratharajan and Murugan, 2018
*Costus speciosus*	PLGA nano-encapsulated	STZ –induced diabetes mellitus	*In vivo* on albino rats	–	↓blood glucose, ↑insulin (I&II) and GLUT4 ↓GLUT2	Alamoudi et al., 2014
*Ficus religiosa*	Solid lipid nanoparticle	STZ and fructose induced –diabetes mellitus	*In vivo* on Wistar rats	Ave: 200 nm	↓ blood glucose ↑insulin level	Priyanka et al., 2018
*Momordica charantia*	Nanoemulsion	ALX-induced diabetes mellitus	*In vivo* on albino rats	<100 nm	↑SOD, ↑GPx, ↓MDA	Paul et al., 2014
*Zingiber officinale*	Green synthesized AgNPs	STZ –induced diabetes mellitus	*In vivo* on Wistar albino rats	123.8 nm	↓ blood sugar	Garg et al., 2016
*Silybum marianum*	Green synthesized ZnO nanoparticle	ALX-induced diabetes mellitus	*In vivo* on Wistar rats	18.8–19.9 nm	↓FBS, TC, TAG ↑insulin, HDL-C	Arvanag et al., 2019
*Nasturtium officinale*	Green synthesized ZnO nanoparticle	ALX-induced diabetes mellitus	*In vivo* on Wistar rats	14 nm	↓FBS, TC, TAG ↑insulin, HDL-C	Bayrami et al., 2019
*Punica granatum*	Green synthesized Au nanoparticle	STZ-induced diabetic nephropathy	*In vivo* on BALB/c mice	20 nm	↓MAPK/NF-κB/STAT3 ↓ RAGE- NOX-4/p47^phox^ ↓ROS ↑Nrf2 ↓renal fibrosis	Manna et al., 2019
*Morus alba*	Green synthesized Ag nanoparticle	Diabetic rats intoxicated with Aluminum	*In vivo* on Albino rats	35 nm	↓deterioration in retinal cell layer ↓ Aluminum and glucose	Xu et al., 2019
*Smilax glabra*	Green synthesized Au nanoparticle	HFD and STZ- induced obese diabetes rats	*In vivo* on Wistar rats	21 nm	↓TNFα and IL-β ↓leptin ↑adiponectin ↓body weight and blood glucose	Ansari et al., 2019

*STZ, streptozotocin; MCT, monocrotaline; PAH, pulmonary arterial hypertension; MDA, malondialdehyde; Ave, average; TC, total cholesterol; TAG, triacylglycerol; LDL-C, low-density lipoprotein-cholestrol; VLDL, very-low-density lipoprotein; HDL-C, high-density lipoprotein; SH, sulfhydryl group; GPx, glutathione peroxidase; SOD, superoxide dismutase; Nrf2, nuclear factor erythroid 2–related factor 2; RV, right ventricular; IL-6, interleukin 6; PDGF, platelet-derived growth factor; FGF, fibroblast growth factors; VEGF, vascular endothelial growth factor; TGFβ, transforming growth factor β; TNFα, tumor necrosis factor; GLUT4, glucose transporter type 4; HbA1c, hemoglobin A1c; PLGA, poly lactic-co-glycolic acid; GK, glucokinase; IRA, insulin receptor A; PKLR, Pyruvate Kinase L/R; ALX, Alloxan; FBS, fasting blood sugar.*

The findings of these studies clearly confirm that most of phytomedicines can be successfully formulated by various nano-delivery approaches and thus successfully delivered to induce the required therapeutic effect. In addition to the proven role of nano-delivery systems, various loading methods, which are also discussed here, seem to be a critical factor. Moreover, targeted delivery of nano-formulated phytomedicines can pave the way to link traditional medicine with modern pharmaceutical techniques to be used in a wide range of diseases, including metabolic disorders.

## Author Contributions

ZN, MH, and ZI did a literature review and prepared the first draft of the manuscript. ZN, RB, and MH edited the manuscript and proposed and included some vital modifications. MF and MA design throughout the work and did the final edition of the manuscript. ZN, MH, MF, and RB revised the manuscript.

## Conflict of Interest

The authors declare that the research was conducted in the absence of any commercial or financial relationships that could be construed as a potential conflict of interest.
